# Antisocial Personality Disorder in Mexican Women: A Study of Sociodemographic Variables and Comorbid Mental Disorders

**DOI:** 10.7759/cureus.80035

**Published:** 2025-03-04

**Authors:** Gala Paulina Sanchez Goddard, Perla Selene Rodriguez Partida, Ismael Aguilar Salas, Héctor Eduardo Guzmán González

**Affiliations:** 1 Department of Education, Ramón de la Fuente Muñiz National Institute of Psychiatry, Mexico City, MEX; 2 Department of Hospitalization, Ramón de la Fuente Muñiz National Institute of Psychiatry, Mexico City, MEX; 3 Department of Geriatrics, Ramón de la Fuente Muñiz National Institute of Psychiatry, Mexico City, MEX

**Keywords:** antisocial personality disorder, female gender, psychiatric comorbidities, sociodemographic variables, women

## Abstract

Introduction: Antisocial personality disorder (APD) is mainly based on social irresponsibility that leads to delinquent, criminal, and exploitative behavior toward others, as well as difficulty in adapting to social norms. This disorder predominates in the male gender, and therefore, most of the research to date has been conducted in men. In the following study, we aimed to describe the main sociodemographic variables and psychiatric comorbidities presented in women with a diagnosis of APD.

Methods: We assessed 54 women with a diagnosis of APD, of whom sociodemographic characteristics were collected, and the Structured Clinical Interview for the Diagnostic and Statistical Manual of Mental Disorders-IV Axis II Disorders (SCID-II) and the Mini-International Neuropsychiatric Interview (M.I.N.I.) 5.0.0 were applied.

Results: We found a weak positive correlation with the obsessive (*r_s_* = 0.28, *p* = 0.039), passive-aggressive (*r_s_*= 0.29, *p* = 0.034), paranoid (*r_s_* = 0.39, *p* = 0.004), narcissistic (*r_s_* = 0.36, *p* = 0.008), and borderline (*r_s_* = 0.35, *p* = 0.010) personality domains, with a median age of 24.00 (IQR = 11.50) years, and the majority mainly being single and unemployed. We also found high rates of suicidal risk, depression, anxiety, and substance abuse, which was consistent with what has been reported elsewhere.

Conclusion: Research on APD and its comorbidities in women is limited, especially in Mexico, where women with APD show similar patterns to those in developed countries. This suggests the need for gender-specific interventions and a dimensional approach to improve the diagnosis and treatment of APD.

## Introduction

Antisocial personality disorder (APD) is primarily characterized by social irresponsibility involving delinquent, criminal, and exploitative behavior toward others, with a total absence of remorse, repeated violation and disregard of the rights of others, and inability to adapt to social norms [1]. An essential characteristic of this diagnosis is that its incidence and prevalence vary depending on the sample studied. In Mexico, according to the National Survey of Psychiatric Epidemiology (2001-2002), the lifetime prevalence of dissocial disorder in men was 10.3% and in women was 2.3% [2]. Other studies suggest that the prevalence of APD in the general population ranges from 1% to 6%, and in prison systems, it may reach up to 80% [3]. Among the main risk factors identified for the development of APD, adverse childhood experiences, child neglect, and being a victim of violence are found [4]. A study conducted in the USA focusing on women with APD found that at least 59.6% of women had a history of violence, with 30.7% experiencing emotional violence, 40% neglect, 19.5% physical violence, and 17.5% sexual abuse. Additionally, 75.5% reported that they had suffered at least two types of maltreatment, and 5% had been exposed to all of them [5]. Likewise, another research in the same country reported that 56.5% of the women assessed never got married, and 52% had less than 12 years of education [6]. An additional difference found in women regarding risk factors is that overall, women tend to have lower scores on the health scales and lower social support, suggesting increased mental health needs [7]. At the genetic level, alterations in the number of tandem copies of the monoamine oxidase A (MAOA) gene, which plays a role in modulating responses to stress, have been observed in women with APD. In a sample of 2,450 American women, participants with high-functioning MAOA and who had suffered childhood maltreatment were at increased risk for antisocial behavior [8]. In terms of the presentation of antisocial behavior, it has been reported that in women with APD, behavioral problems occur at later ages, they have fewer encounters with the law, and they score higher in the subscales of sexual and psychological abuse on the Childhood Trauma Questionnaire compared with men [6].

APD is more commonly diagnosed in men, and most of the research has primarily focused on male populations and in developed countries, mainly in the USA. As a result, there is limited information available on APD in Mexican women. However, the few studies that have specifically examined women with APD in other countries have revealed significant gender differences in terms of risk factors, sociodemographic characteristics, molecular changes, and the presentation of antisocial behaviors. In this context, our research aims to explore APD in Mexican women to identify key psychiatric comorbidities and sociodemographic variables associated with the disorder. To our knowledge, no study has been conducted on this subject within this population. Thus, we believe it is crucial to address this gap to gain a deeper understanding of APD from a gendered perspective in Mexico.

## Materials and methods

Patients

The study was conducted prospectively between August 2022 and December 2023 with individuals receiving care at the Ramón de la Fuente Muñiz National Institute of Psychiatry, a leading reference center for mental health in Mexico City and one of the hospitals serving the largest psychiatric population in the country. The inclusion criteria were women aged 18 years and older who met the diagnosis of APD according to the Diagnostic and Statistical Manual of Mental Disorders (DSM), Fifth Edition. Exclusion criteria included participants with a diagnosis of a psychotic spectrum disorder or the presence of hypomania/mania, as evidenced by the Mini-International Neuropsychiatric Interview (M.I.N.I.) 5.0.0. A trained psychiatrist collected sociodemographic and clinical characteristics (personality and psychiatric comorbidities assessment) in a 60-minute session.

Personality assessment

For the personality assessment, we used the Structured Clinical Interview for DSM-IV Axis II Disorders (SCID-II) for its international validity. This is a structured clinical interview used to assess personality disorders according to the criteria of the DSM-IV. This tool helps mental health professionals diagnose personality disorders such as antisocial, borderline, and narcissistic, among others. In the validation of its Spanish version, the internal consistency of this scale was determined using the Kuder-Richardson coefficient. The results showed that the antisocial, schizotypal, avoidant, narcissistic, and paranoid personality disorders obtained an acceptable correlation. In the case of antisocial personality disorder, the Kuder-Richardson coefficient was 0.75 [9].

Psychiatric assessment

The M.I.N.I. 5.0.0 is a structured interview adapted to the diagnostic criteria of disorders included in the International Classification of Diseases, Tenth Revision (ICD-10) and DSM-IV. It is a quick and easy instrument to apply. An average application time of 15 minutes has been estimated. The author of the original instrument included 19 disorders with a 12-month prevalence of 0.5% (17 axis I disorders, one suicidality module, and one axis II disorder). Good to very good kappa values ​​have been identified when comparing the M.I.N.I. 5.0.0 with the Structured Clinical Interview for DSM-III (SCID-P), except for the section that evaluates current dependence on other drugs (kappa < 0.50). Interrater reliability with a kappa value > 0.70 has been calculated for all included disorders [10].

Sample size

To determine the sample size, the number of women who attended the Ramón de la Fuente Muñiz National Institute of Psychiatry in 2021-2022 was identified, obtaining a total of 22,731 women. Considering the prevalence of Mexican women with APD (2.3%), the proportion estimation formula was used with a confidence interval of 95% and a precision level of 4%, giving a sample of 54 participants [2].

Statistical analysis

Categorical variables are represented as frequencies and percentages, while continuous variables are expressed as medians with interquartile ranges (IQR). The Shapiro-Wilk test was employed to assess the normality of the data. Since the Shapiro-Wilk test indicated a lack of normality, a Spearman’s rank correlation was conducted to assess the relationship between variables. The statistical significance was established at *p* < 0.05. Statistical analyses were performed with Jamovi 2.3.28.

The study was approved by the Ethics Committee of the Ramón de la Fuente Muñiz National Institute of Psychiatry with the approval number CEI/C/041/2022. All the patients provided their written informed consent to participate in this study. To protect the confidentiality of participants, personal data were registered with an alphanumeric code. The authors have no conflicts of interest to declare.

## Results

Sociodemographic characteristics

The sample comprised 54 Mexican women. A total of 30 (55.60%) and 24 (44.40%) women were recruited from the inpatient and outpatient services, respectively. The median age was 24.00 (IQR = 11.50) years, of whom 47 (87.03%) were single, and 31 (57.40%) were unemployed. The median educational level was 12.00 (IQR = 5.75) years.

SCID-II domain scores

The sample’s median score for the antisocial domain was 7.00 (IQR = 4.00). Table 1 summarizes the rest of the scores.

**Table 1:** Scores obtained from the application of SCID-II. SCID-II = Structured Clinical Interview for the Diagnostic and Statistical Manual of Mental Disorders-IV Axis II Disorders; IQR = interquartile range.

Parameters	Median	IQR
Avoidant	3.00	3.50
Dependent	2.00	3.00
Obsessive	3.00	3.00
Passive-aggressive	5.00	2.00
Dysthymic	6.00	3.00
Paranoid	4.50	3.75
Schizotypal	2.00	3.75
Schizoid	2.00	2.00
Histrionic	3.00	3.00
Narcissistic	7.00	4.75
Borderline	13.00	3.00
Antisocial	7.00	4.00

Psychiatric assessment

Based on the M.I.N.I. 5.0.0, we identified 35 (64.81%) participants with suicidality, 33 (61.11%) with major depressive disorder, 26 (48.15%) with generalized anxiety disorder (GAD), 22 (40.74%) with other substance dependence/abuse, and 21 (38.89%) with alcohol dependence/abuse. The rest of the clinical characteristics are shown in Table 2.

**Table 2:** Clinical characteristics of the sample based on the M.I.N.I. 5.0.0. M.I.N.I. 5.0.0=Mini-International Neuropsychiatric Interview 5.0.0; OCD = obsessive-compulsive disorder; PTSD = post-traumatic stress disorder.

Clinical characteristics	Number	Percent
Major depressive disorder	33	61.11
Dysthymia	15	27.78
Suicidality	35	64.81
Panic disorder	16	29.63
Agoraphobia	3	5.56
Social phobia	5	9.26
OCD	0	0.00
PTSD	3	5.56
Alcohol dependence/abuse	21	38.89
Other dependence/abuse	22	40.74
Anorexia nervosa	1	1.85
Bulimia nervosa	11	20.37
Generalized anxiety disorder	26	48.15

Correlation of antisocial domain score with the rest of the domains assessed by the SCID-II

**Figure 1:**
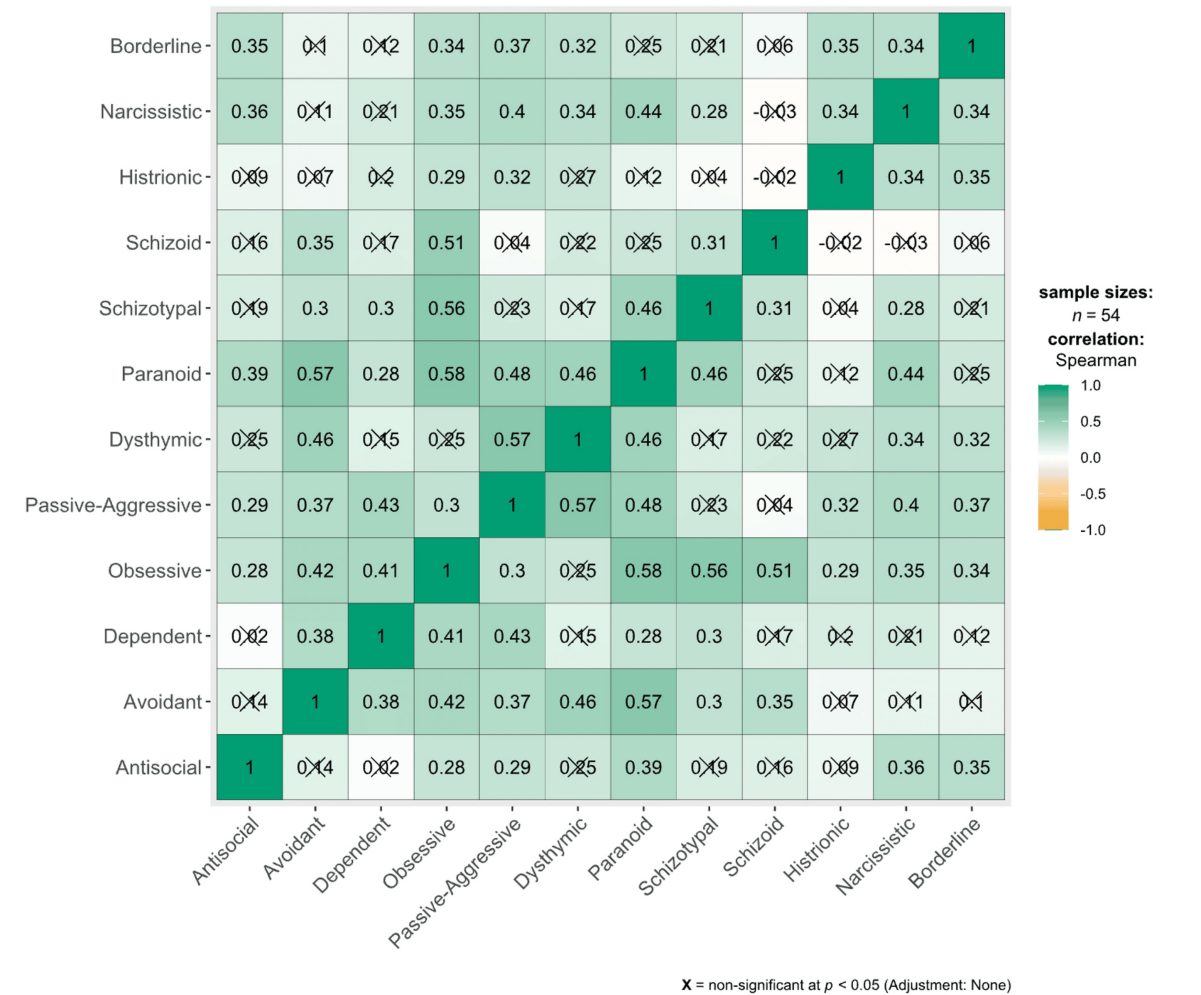
Heat map correlating the domains assessed by the SCID-II. SCID-II = Structured Clinical Interview for the Diagnostic and Statistical Manual of Mental Disorders-IV Axis II Disorders.

A Spearman’s rank correlation was conducted to assess the relationship between the antisocial domain score and the other domains evaluated by the SCID-II. A weak positive correlation was identified between the antisocial and obsessive (*r_s_* (52) = 0.28, *p* = 0.039), passive-aggressive (*r_s_* (52) = 0.29, *p* = 0.034), paranoid (*r_s_* (52) = 0.39, *p* = 0.004), narcissistic (*r_s_* (52) = 0.36, *p* = 0.008), and borderline (*r_s_* (52) = 0.35, *p* = 0.010) domains (Figure 1).

Criminal conduct and antisocial behavior

Regarding past criminal conduct and antisocial behavior, 13 (24.07%) belonged to gangs that caused disturbances, 45 (83.33%) defied authority, 31 (57.41%) stole goods and/or money, and only one (1.85%) trespassed private property.

## Discussion

APD in women and sociodemographic characteristics

Regarding the objective of analyzing the sociodemographic characteristics, we identified 31 (57.40%) out of our 54 participants as unemployed. This is congruent with another study of American women with APD that reported that at least 34 (74.10%) had difficulties maintaining their jobs. In this same study, the mean age at presentation was 27.2 years, as we identified in our study, where the median age was 24.00 years [11]. Concerning education level, we identified a median of 12.00 years of formal education, similar to what has been reported elsewhere. In a study from the USA, where the sample consisted of 70 women with a diagnosis of APD, they found a mean of 13.76 years of formal education [6]. This suggests that women with APD might be less likely to attend university or have professional careers, which could also be associated with low levels of employment and low socioeconomic income.

APD in women and other comorbid personality disorders

Among the findings obtained regarding other comorbid personality disorders, a weak positive correlation was found with obsessive, passive-aggressive, paranoid, narcissistic, and borderline personality disorders. This is in line with the current literature, which has identified that women diagnosed with APD are comorbid with borderline and narcissistic personality disorders in 28.70% and 19.79%, respectively. Additionally, the presence of comorbid obsessive and paranoid personality disorders was identified in 26.97% and 29.52%, respectively [7]. A key factor that could explain the association between borderline personality disorder (BPD) and APD is impulsivity [12]. This is considered a transdiagnostic symptom among many psychiatric illnesses and, due to its impact, has been studied by different authors. For example, in a study that included 692 participants, 286 women between the second and third decade of life and with a diagnosis of both APD and BPD had a greater tendency to impulsivity compared with healthy controls [13]. Similarly, another study focused on assessing impulsivity with a transdiagnostic and longitudinal approach found a positive association between impulsivity and hostility, as well as physical and verbal aggression, all factors found in APD [14].

Psychiatric comorbidities

We identified several psychiatric comorbidities in our sample, mainly major depressive disorder, GAD, and alcohol and other substance dependence/abuse. Of our participants, 33 (61.11%) met the criteria for depression, which is in line with a prevalence of depression in women with APD that has ranged from 41.1% to 63.4% [15,16]. Similarly, GAD, which was identified in 26 (48.15%) of our participants, has also been reported to be frequent in this population. For example, a study conducted in the United Kingdom that assessed 3,147 women with APD found that up to 22.7% had comorbid GAD. In this study, the authors also described that this diagnosis was associated with an increased risk of having a comorbid substance use disorder [17]. Other studies have also identified a high prevalence of anxiety disorders. For example, the National Comorbidity Survey from the USA, which included a sample of 5,877 participants, found that at least 54.3% of the participants with APD had an anxiety disorder [18]. Another study identified that 34.13% of the women with ADP had anxiety disorders [7]. These results suggest that despite the inherent characteristics of ADP, women with this diagnosis are at risk of developing affective and anxiety disorders. Additionally, our results showed that 21 (38.89%) had alcohol dependence/abuse, and 22 (40.74%) had other substance dependence/abuse. Although most of the literature focused on comorbidity between APD and substance use is in men, some studies focused on women have also found an association between APD and substance use. For example, one study that included 41 women with APD found that 56.1% had alcohol dependence, and 70.7% had a dependence on at least one other substance [16].

In addition to studying other psychiatric comorbidities, we considered it essential to assess the relationship between APD and suicidal risk, as having more than one psychiatric diagnosis proportionally increases this risk [19]. Although our results identified 35 (64.81%) participants who had suicidal risk, this is considerably higher than what has been reported elsewhere. In a Brazilian study with a sample of 3,781, of which 41 participants had APD, the prevalence of suicide risk in women was up to 3.2% [20]. This could be explained by the fact that many of the participants included in this study were from the inpatient area. However, it would be necessary to address this issue further to determine the association between APD and suicidal risk.

Criminal behavior

A fact that is well established in literature is that the male gender is involved in antisocial behavior more often than women. However, when considering the whole dimension of antisocial behavior, the gender gap narrows significantly. For example, in one study, it was reported that violence against partners was the same among both genders [21]. One difference reported between men and women is the age at which antisocial behavior appears. For example, in women, it occurs between the ages of 12 and 14, while in boys, it tends to be more prevalent between 10 and 13 years [22]. In addition, some authors proposed a four-category model that encompasses different categories for antisocial behavior: (1) overt behavior, which consists of interpersonal violence; (2) covert behavior, like property crimes; (3) conflicts with authority, such as defiant and avoidant behaviors; (4) reckless behaviors, such as risky sexual activity and substance use/abuse. In women, it has been seen that they tend to have more covert behavior and risky sexual activity than men [23]. Regarding criminal behavior in our study population, it was found that the most prevalent antisocial behavior was defying authority, followed by stealing and belonging to gangs that cause disturbances. These findings are consistent with the literature since significant differences have been seen in terms of manifestations of APD depending on gender [24].

Limitations and strengths

The first limitation of our study is that since this was conducted in a specialized psychiatric hospital focused on offering treatment to people with severe psychiatric disorders, it likely excluded people with a single personality disorder. This meant that when applying the SCID-II and the M.I.N.I. 5.0.0, our participants, unlike the general population, were more likely to have more than one psychiatric disorder and suicide risk at the time of the interview, so our results might not be representative of the general population. The second limitation is the absence of a group of Mexican men with a diagnosis of APD to compare the studied variables. It will be necessary for future studies to include this group to identify more significant differences in terms of gender. The third limitation is the number of women included in the study. Increasing the sample size to improve the statistical power will be necessary.

Regarding the main strength of this study, we consider the fact that, for the first time, a research protocol for APD was carried out exclusively on Mexican women. Also, we evaluate not only the sociodemographic characteristics and the comorbidities of this disorder but also the form of presentation of the antisocial behavior because, as it is seen in the literature, this type of behavior in women tends to be less externalized than in men, which makes the identification of this entity difficult. For this reason, we believe that this type of study may allow us to consider a different approach and assessment of APD in Mexican women, and this may give us better ways of dealing with and orienting this population. According to the current literature, antisocial behavior in females may not be as overt in terms of direct aggression or externalized behaviors compared with men. However, it is essential to consider covert antisocial behaviors within the antisocial spectrum, as these issues are not usually explicitly explored or detailed in the current diagnostic criteria of the most frequently used scales that explore personality disorders. To our knowledge, there are no current scales focused on APD that encompass the complexity and heterogeneity of antisocial behavior, much less focused on females. Because of this, we consider these types of studies could be used to develop better diagnostic tools, which can help us create better treatment plans.

## Conclusions

Research on APD and its comorbidities in women is limited, especially in developing countries like Mexico, since most of the studies have been carried out in the USA. Our study focusing on Mexican women with APD found patterns like those observed in developed countries, such as being single, unemployed, and in their 20s. The high prevalence of mood, anxiety, and substance use disorders in this population probably contributed to a significant risk of suicidal behavior. These findings highlight the need for more focused research and a better understanding of this disorder in diverse populations. The study also revealed specific antisocial behaviors, with women tending to exhibit covert behaviors such as defying authority rather than overt aggression seen in men. This suggests that interventions for women with APD should be tailored to address these unique behaviors, mainly the early detection and initiation of therapy focused on social reintegration. The research emphasizes the need for a dimensional approach to APD, accounting for its heterogeneity and improving diagnosis and treatment. Such an approach would better address the varied presentations of APD and offer more effective preventive measures. Ultimately, the study calls for improved strategies to reduce risks and enhance outcomes for women with APD.
